# Alisol A attenuates high‐fat‐diet‐induced obesity and metabolic disorders via the AMPK/ACC/SREBP‐1c pathway

**DOI:** 10.1111/jcmm.14380

**Published:** 2019-05-29

**Authors:** Chiakang Ho, Ya Gao, Danning Zheng, Yanjun Liu, Shengzhou Shan, Bin Fang, Yixuan Zhao, Dingzhong Song, Yifan Zhang, Qingfeng Li

**Affiliations:** ^1^ Department of Plastic & Reconstructive Surgery Shanghai Ninth People’s Hospital, Shanghai Jiao Tong University School of Medicine Shanghai China; ^2^ China State Institute of Pharmaceutical Industry, National Pharmaceutical Engineering Research Center Shanghai China

**Keywords:** alisol A, AMPK/ACC/SREBP‐1c, hepatic steatosis, metabolism dysfunction, obesity

## Abstract

Obesity and its associated metabolic disorders such as diabetes, hepatic steatosis and chronic heart diseases are affecting billions of individuals. However there is no satisfactory drug to treat such diseases. In this study, we found that alisol A, a major active triterpene isolated from the Chinese traditional medicine Rhizoma Alismatis, could significantly attenuate high‐fat‐diet‐induced obesity. Our biochemical detection demonstrated that alisol A remarkably decreased lipid levels, alleviated glucose metabolism disorders and insulin resistance in high‐fat‐diet‐induced obese mice. We also found that alisol A reduced hepatic steatosis and improved liver function in the obese mice model.In addition, protein expression investigation revealed that alisol A had an active effect on AMPK/ACC/SREBP‐1c pathway. As suggested by the molecular docking study, such bioactivity of alisol A may result from its selective binding to the catalytic region of AMPK.Therefore, we believe that Alisol A could serve as a promising agent for treatment of obesity and its related metabolic diseases.

## 1 INTRODUCTION

Obesity has undoubtedly become a global concern and affects more than 2.1 billion individuals worldwide.^1^ This disease not only compromises cosmetic appearances but also negatively impacts multi‐organ functions.^2^ Current therapeutic approaches to obesity include exercise, dietary control, bariatric surgery and anti‐obesity drugs.^3^, ^4^, ^5^ Nevertheless, exercise and dietary control appear to be too difficult to implement for a long period, while bariatric surgery may cause noticeable complications and side effects.^6^ And several drugs, including orlist, sibutramine and lorcaserin, are used in the clinic to treat obesity. Worrying is that several of these chemical drugs have unacceptable side effects^7^ like steatorrhea, dry mouth, constipation, headache and insomnia. So it is recommended these drugs only be prescribed when benefits of the treatment outweigh the risks. Therefore, it is of great importance to explore novel anti‐obesity agents with less side effets and better efficacy.

Numerous Chinese herbs have been used traditionally for the treatment of obesity and its accompany symptons, including Coptidis Rhizome (Huanglian),^8^, ^9^, ^10^ Crataegi Fructus (Shanzha)^11^, ^12^ and Ginseng Radix et Rhizoma (Renshen).^13^, ^14^ While Rhizoma Alismatis (Zexie), which also been used for obesity related syndrome receiving relatively little attention than its famous counterparts. Rhizoma Alismatis, the dried rhizome of *Alisma orientale*, its effects mentioned in Chinese herbal medicine books are promoting urination to drain dampness, discharging heat and resolving turbidity and lowering lipid.^15^, ^16^ With the deepening of modern medical research, it has been more widely studied.

Modern medical researches about this herb found active ingredients in it and numerous studies have shown that Rhizoma Alismatis extract has various biological activities, such as hypolipemic,^17^ antidiabetic,^18^ anti‐urinary stone,^19^ anti‐inflammation^20^ and anti‐tumour^21^ activities. Li S et al found that Rhizoma Alismatis extract can significantly reduce the serum LDL levels of ApoE‐/‐ mice and promote the removal of chylomicron in the liver.^22^ Additionally, researchers demonstrated that alisol acetates inhibited the activity of HMG‐CoA reductase and subsequently lowered the TC, TG and low‐density lipoprotein cholesterol concentrations and raised high‐density lipoprotein cholesterol concentrations in hyperlipidemic mice.^23^ Alisol A 24‐acetate, an active triterpene isolated from Rhizoma Alismatis, can ameliorate steatohepatitis by inhibiting oxidative stress and stimulating autophagy through the AMPK/mTOR pathway.^24^ In addition, alisol A 24‐acetate could also decrease the number of lipid droplets in HepG2 cells, thus preventing hepatic steatosis.^25^ However, the effect of alisol A, a main ingredient in Rhizoma Alismatis extract, on obesity and metabolic dysfunctions has not yet been investigated.

In our study, we evaluated the therapeutic efficiency and toxicity of alisol A in high‐fat‐diet‐fed mice and demonstrated that alisol A is safe for use and helps decrease body weight with improve hyperlipidemia and glycometablism, it aslo alleviated liver steatosis in an obese mouse model. Furthermore, the AMP‐activated protein kinase (AMPKα)/acetyl‐CoA carboxylase (ACC) pathway seems to be involved in the anti‐obesity effects of alisol A.

## 2 MATERIALS AND METHODS

### 2.1 Reagents

Alisol A (purity >99%) was obtained from the China State Institute of Pharmaceutical Industry, National Pharmaceutical Engineering Research Center (batch number: W20151210, Shanghai, China). Rimonabant (purity >98%) was purchased from Xi'an zelang Biological Technology Co., Ltd. (batch number: XAZL170213‐1, Xi'an, China). All other reagents were of analytical grade.

### 2.2 Animal study

C57BL/6 mice 6 weeks of age were purchased from Shanghai Super‐B&K Laboratory Animal Company (Shanghai, China) and housed under standard conditions approved by the Shanghai Jiao Tong University Animal Care and Use Committee.

Mice were randomly divided into two groups: a control group (n = 16) fed a normal diet (D12450B, Hua Fu Kang, Beijing, China) with 10% of kcals from fat and an obesity group (n = 48) fed a high‐fat diet (D12492, Hua Fu Kang, Beijing, China) with 60% of kcals from fat. After 5 weeks of high‐fat diet feeding, mice in the obesity group were randomly divided into three groups: the high‐fat (HF) diet group, the high‐fat diet with rimonabant (HFR; 40 mg/kg/day) group and the high‐fat diet with alisol A (HFA; 100 mg/kg/day) intraperitoneal injection once a day for 4 weeks group. Alisol A or rimonabant was dissolved in Cremophor EL solution (vehicle). Both lean control and high‐fat diet control mice were also given equal amounts of the vehicle (200 µL). At the end of all experiments, mouse blood was collected from the retro‐orbital plexus and centrifuged for 15 minutes (1200 *g*). Serum samples were collected for biochemical parameter determination. After taking the blood, the mice were sacrificed for liver/adipose tissue/skeletal muscle tissues harvesting, and the body weight and abdominal fat weight were measured.

### 2.3 Histology, immunohistochemistry and Oil Red O staining

Liver and adipose tissues were fixed with 4% PFA, embedded in paraffin and cut into 5‐μm‐thick sections. H&E staining was performed according to the manufacturer's protocol. Frozen liver tissues were sectioned (5 μm thick) using a freezing microtome (Leica Microsystems, Buffalo Grove, IL). As for immunohistochemistry, sections were incubated with primary antibody against F4/80 (Abcam, Cambridge, UK, 1:100) dilution diluted in blocking solution overnight at 4°C. After being incubated with HRP‐conjugated secondary antibody, the sections were counterstained with hematoxylin and developed with diaminobenzidine. Sections were stained with Oil Red O and counterstained with haematoxylin to detect hepatic lipid accumulation.

### 2.4 RNA purification and quantitative real‐time PCR

The total RNA was isolated using TRIzol reagent (Invitrogen, Carlsbad, CA). RT‐qPCR was performed with an ABI 7900HT system using SYBR Premix (Takara, Dalian, China) according to the manufacturer's instructions. Glyceraldehyde 3‐phosphate dehydrogenase (GAPDH) was used as an internal control. The primers used in this study were as follows: GAPDH: forward, 5′‐AGGTCGGTGTGAACGGATTTG‐3′; reverse, 5′‐TGTAGACCATGTAGTTGAGGTCA‐3′; TNF‐α: forward, 5′‐CCCTCACACTCAGATCATCTTCT‐3′; reverse, 5′‐GCTACGACGTGGGCTACAG‐3′; IL‐1β: forward, 5′‐GCAACTGTTCCTGAACTCAACT‐3′; reverse, 5′‐ATCTTTTGGGGTCCGTCAACT‐3′; IL‐6: forward, 5′‐TAGTCCTTCCTACCCCAATTTCC‐3′; reverse, 5′‐TTGGTCCTTAGCCACTCCTTC‐3′; IL‐8: forward, 5′‐CAAGGCTGGTCCATGCTCC‐3′; reverse, 5′‐TGCTATCACTTCCTTTCTGTTGC‐3′; Fasn: forward, 5′‐GGAGGTGGTGATAGCCGGTAT‐3′; reverse, 5′‐TGGGTAATCCATAGAGCCCAG‐3′; CAT: forward, 5′‐GCTGCCAGAACCGTGGTAAA‐3′; reverse, 5′‐CCTTGAGGTAATAGTCCAGGGA‐3′; CPT2: forward, 5′‐CAGCACAGCATCGTACCCA‐3′; reverse, 5′‐TCCCAATGCCGTTCTCAAAAT‐3′; Echs1: forward, 5′‐AGCCTGTAGCTCACTGTTGTC‐3′; reverse, 5′‐ATGTACTGAAAGTTAGCACCCG‐3′.

### 2.5 Serum biochemistry assays and blood glucose measurements

Serum concentrations of alanine aminotransferase (ALT), aspartate aminotransferase (AST), creatinine (Cr), urea nitrogen, triglycerides (TG), total cholesterol (TC), low‐density lipoprotein cholesterol (LDL‐C) and high‐density lipoprotein cholesterol (HDL‐C) were determined using an automatic biochemical analyser (Hitachi Auto Analyser 7170, Japan).

Serum concentrations of insulin and FABP4 were determined by sandwich ELISA. The primary antibodies were as follows: anti‐insulin and anti‐FABP4 (Abcam, Cambridge, UK). The absorbance was determined at 450 nm on a microplate reader (Tecan Group AG, Männedorf, Switzerland). The level of nonesterified fatty acids (NEFAs) was detected using the Free Fatty Acid Assay Kit (Abcam, Cambridge, UK) according to the manufacturer's instructions.

Mouse blood was dropped onto a glucose test strip (Accu‐Check Active, Roche) and measured by a glucometer (Accu‐Check Active, Roche). For the glucose tolerance test (IP‐GTT), mice were given an intraperitoneal injection (ip) of 100 mg/mL D‐glucose (2 g/kg body weight) after overnight fasting, and blood glucose concentrations were monitored. For the insulin tolerance test (IP‐ITT), mice were fasted for 4 hours before ip administration of human insulin (0.75 U/kg body weight, Santa Cruz Biotechnology, Dallas, TX), and blood glucose concentrations were monitored.

### 2.6 Oral fat tolerance test

For the OFTT, overnight fasted mice were given an olive oil load (10 ml/kg, orally), and all the intervention injections were performed 30 minutes before the oil was given. Mice were mildly anaesthetized using isoflurane anaesthesia, and blood was collected from the retro‐orbital plexus at different time points. The plasma was separated by centrifugation, and the plasma triglyceride level was measured by using an automatic biochemical analyser (Daytona, Randox Inc UK).

### 2.7 Western blotting

Tissues and cultured cells were lysed with RIPA buffer supplied with protease inhibitor cocktail (Roche, Mannheim, Germany). Concentrations of protein were detected by the bicinchoninic acid (BCA) assay (Thermo Fisher Scientific). Total protein extract (20 μg) was separated by 8% or 10% sodium dodecyl sulfate‐polyacrylamide gel electrophoresis and transferred onto polyvinylidene difluoride membranes (Millipore, Bedford, MA). The primary antibodies were as follows: anti‐AMPK, anti‐p‐AMPK, anti‐ACC, anti‐p‐ACC and anti‐SREBP‐1c (1:1000; Cell Signalling Technology, Beverly, MA). Immunoreactive bands were quantitatively analysed with ImageJ software.

### 2.8 Molecular docking

The crystal structure of the complex of AMPK kinase was downloaded from RCSB Protein Data Bank (PDB ID：5ufu) and prepared by SYBYL‐X 2.0. The docking study was performed using the Surflex‐Dock GeomX (SFXC) in SYBYL‐X 2.0. The binding interaction were generated using PyMOL.

### 2.9 Statistical analysis

The results are expressed as the mean ± standard deviation (SD). To determine significant differences between groups, a one‐way ANOVA with the Bonferroni post hoc test was performed. The statistical analyses were performed with spss 20.0 (SPSS, Chicago, IL), and *P* < 0.05 were regarded as significant.

## 3 RESULTS

### 3.1 Alisol A attenuated high‐fat‐diet‐induced obesity

**Figure 1:**
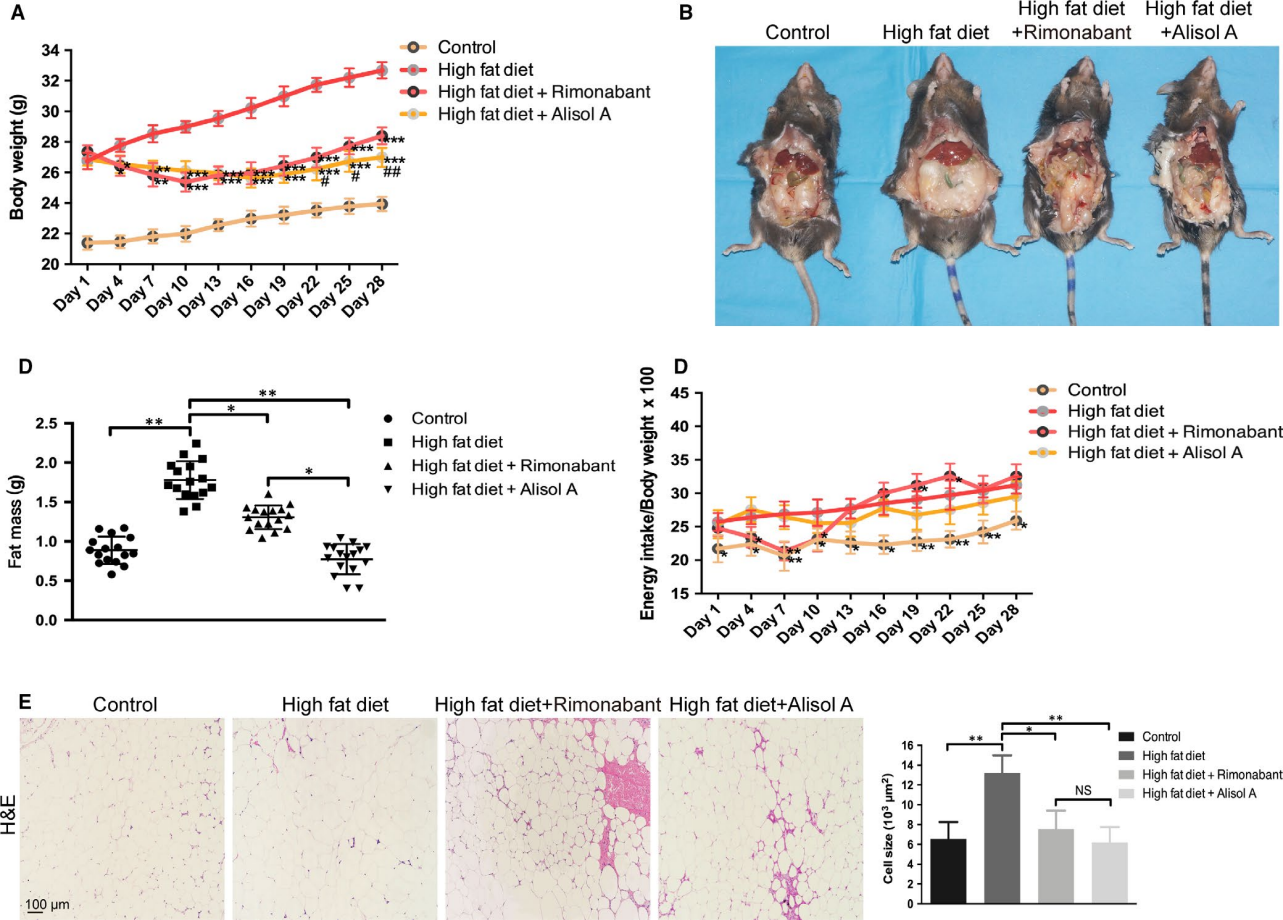
Alisol A attenuates high‐fat‐diet‐induced obesity. A, Body weights of the four groups of mice, including the control group (normal diet), the HF group (high‐fat diet), the HFR group (high‐fat diet with rimonabant intervention) and the HFA group (high‐fat diet with alisol A intervention). B, Representative images of the abdominal cavity and fat pat. C, Weight of dissociated abdominal adipose tissue. D, Difference in the feed consumption of mice in each group during the experiment. E, H&E staining images of abdominal fat tissue and cell size quantification. Data are the mean ± SD. NS = not significant; *P < 0.05, **P < 0.01, ***P < 0.001 vs HF group, #*P* < 0.05, ##*P* < 0.01 vs HFR group

To investigate the effect of alisol A (Figure S1) on obesity, high‐fat‐diet‐induced obese mice were injected with alisol A at a dose of 100 mg/kg/d. Rimonabant, also known as SR141716, a selective CB1 antagonist, was used as a positive control of anti‐obesity for alisol A. Compared to the vehicle‐treated group (the HF group), the alisol A‐treated mice (the HFA group) and the rimonabant‐treated mice (the HFR group) exhibited markedly decreased body weights at all examined time points (Figure 1A). In addition, the body weight was reduced more pronouncedly in the HFA group than in the HFR group between days 19 and 28 (Figure 1A). Consistent with the effects on body weight, the weight of abdominal fat was also significantly decreased in the HFA group and HFR group (Figure 1B,C), and alisol‐A‐treated mice exhibited lower abdominal fat weight than the rimonabant‐treated mice (Figure 1B,C). We then used Energy intake/Body weight (relative food intake) to demonstrate the differences in food intake of each group. Unlike HFA group had fluctuation in relative food intake, HFA group had no significant difference comparing the HF group. (Figure 1D). Furthermore, H&E staining of the abdominal fat of the mice in each group showed that the average size of adipocytes in the HFA group and the HFR group was significantly different than that of the HF group (Figure 1E). These results indicated that although alisol A did not affect relative food intake, it attenuated high‐fat‐diet‐induced obesity in mice.

### 3.2 Alisol A improved lipid and glucose metabolism in high‐fat‐diet‐induced obese mice

**Figure 2:**
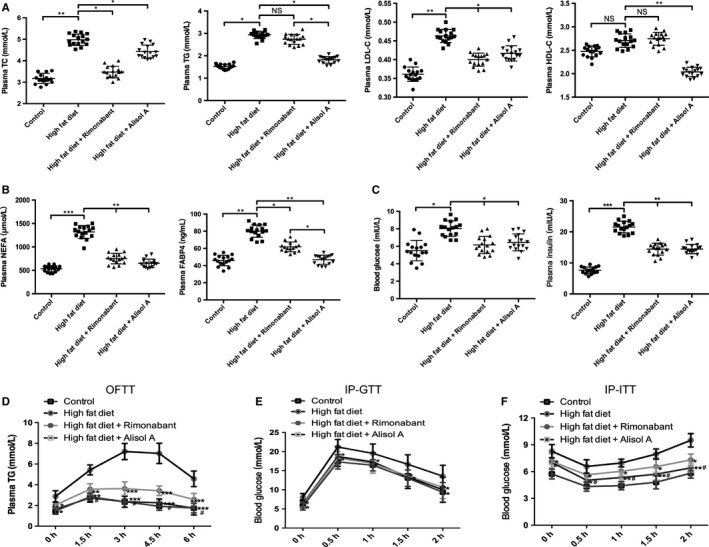
Alisol A improves lipid and glucose metabolism in high‐fat‐diet‐induced obese mice. A, Plasma TG, TC, LDL‐C and HDL‐C in the four groups of mice. B, Plasma levels of NEFA and FABP4 in each group. C, Blood glucose and insulin levels in each group. Data are the mean ± SD. NS = not significant; *P < 0.05, **P < 0.01, ***P < 0.001. D, Plasma TG level during OFTT. Blood glucose concentration during the IP‐GTT (E) and IP‐ITT (F). Data are the mean ± SD. *P < 0.05, **P < 0.01, ***P < 0.001 vs HF group, #*P* < 0.05 vs HFR group

Because of alisol A's excellent performance in DIO mice weight loss, we then evaluated the metabolic profiles. Alisol A reversed the increase of total cholesterol (TC), triglycerides (TG) and LDL‐C caused by obesity (Figure 2A). The increase in the free fatty acids (NEFAs) and free acid binding protein 4 (FABP4) caused by the high‐fat diet could also be reversed by alisol A (Figure 2B). Alisol A not only decreased the random blood sugar but also showed an effect on glycometabolism improvement by reversing the increase in insulin induced by a high‐fat diet (Figure 2C). To investigate the effect of alisol A on intestinal lipid absorption, an oral fat tolerance test (OFTT) was conducted. Treatment with alisol A and rimonabant significantly decreased the elevated plasma triglyceride levels at all examined time points compared to the plasma triglyceride levels of the vehicle‐treated HF group, and the reduction effect of alisol A was more notable than that of rimonabant (Figure 2D). Then, IP‐GTT and IP‐ITT assays were performed to evaluate the effect of alisol A on glucose metabolism. The IP‐GTT and IP‐ITT demonstrated that mice in the HFA had improved glucose tolerance and insulin sensitivity than the HF group and HFR group (Figure 2E‐F).

### 3.3 Alisol A reduced hepatic steatosis in HFD‐induced obese mice

**Figure 3:**
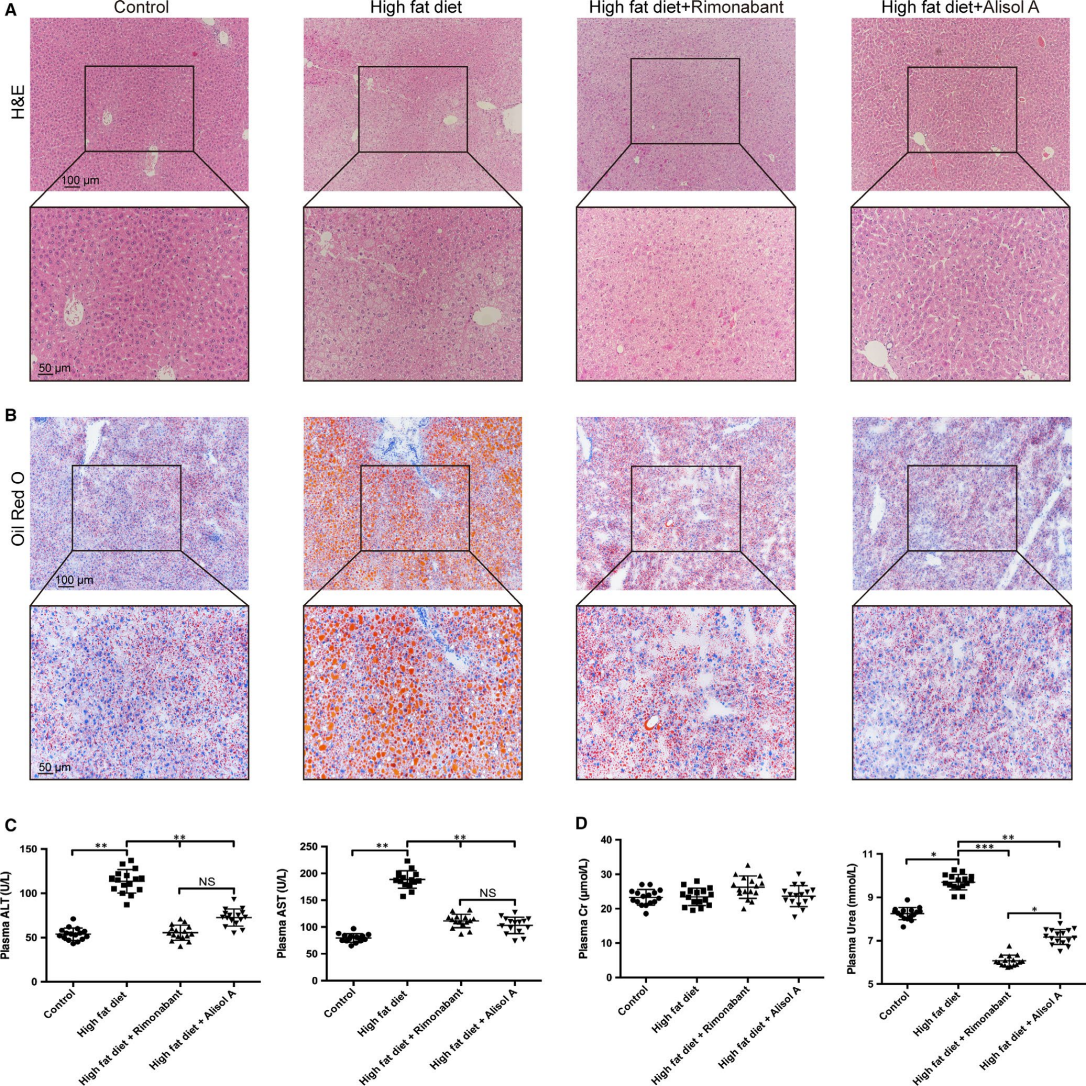
Alisol A rescues high‐fat‐diet‐induced liver steatosis. Representative images of H&E (A) and Oil Red O (B) stained liver sections. Scale bars, 100 μm. Zoom scale bars: 50 μm. C, Plasma levels of ALT and AST in the four groups of mice. D, Plasma levels of creatinine and urea in the 4 groups of mice. Data are the mean ± SD. NS = not significant; *P < 0.05, **P < 0.01, ***P < 0.001

H&E staining showed a number of empty fat vacuoles that were either larger or smaller than the hepatocyte nucleus, known as macrovesicular and microvesicular steatosis, in the HF group (Figure 3A). Both the macrovesicular and microvesicular steatosis decreased substantially after treatment with alisol A as well as rimonabant (Figure 3A). Consistent with the H&E staining data, Oil Red O staining suggested decreased lipid deposition in the HFA and HFR groups (Figure 3B). Moreover, the suppressive effects of alisol A were more notable compared with those of rimonabant according to our histology assay (Figure 3A,B). In addition, biochemical detection was performed to test hepatic and renal function in each group (Figure 3C). A high‐fat diet significantly increased plasma ALT, AST and urea. These alterations could be reversed in the HFA and HFR groups (Figure 3C).

### 3.4 Alisol A alleviated HFD‐induced inflammation in adipose tissue of obese mice

**Figure 4:**
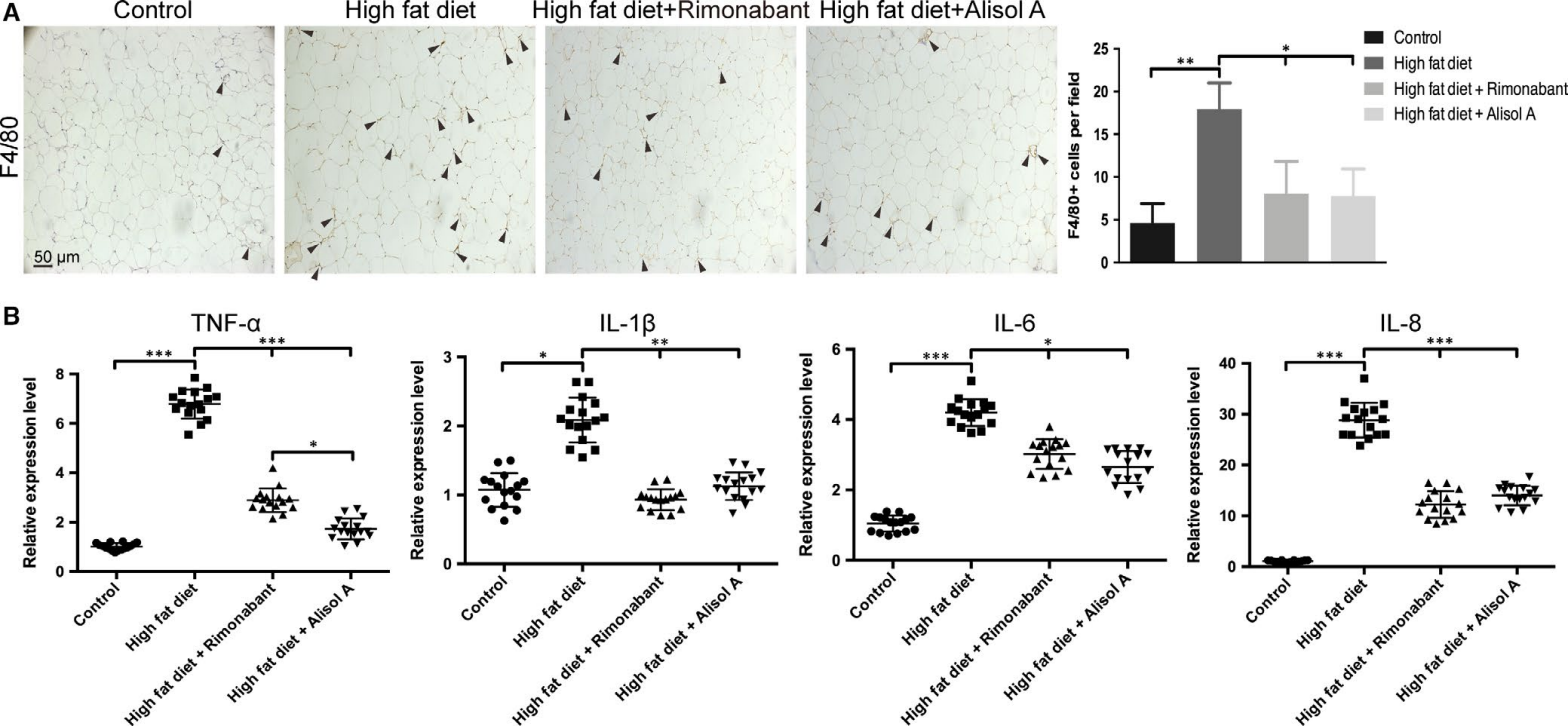
Alisol A alleviated HFD‐induced inflammation. A, Representative images of immunohistochemistry of F4/80 and analysis of expression level in mice adipose tissue. Scale bar = 50 μm. B, Relatively RNA expression and showing levels of TNF‐α, IL‐1β, IL‐6 and IL‐8 in extract of different group of mice adipose tissue. Data are the mean ± SD. NS = not significant; *P < 0.05, **P < 0.01, ***P < 0.001

Given that adipose tissue inflammation and macrophage recruitment is important in the development of insulin resistance in obesity,^26^ we investigated the impact of alisol A on the inflammation and macrophage recruitment. Immunohistochemistry for F4/80, a marker of macrophages, showed that alisol A treatment decreased the HFD‐induced macrophage infiltration in mouse epididymal WAT (Figure 4A). What's more, the TNF‐α, IL‐1β, IL‐6 and IL‐8 mRNA levels (pro‐inflammatory cytokines) were down‐regulated due to alisol A treatment (Figure 4B). These results indicate that alisol A treatment reduced macrophage recruitment and inflammation in adipose tissue of obese mice.

### 3.5 Alisol A enhanced lipolysis in different tissues of obese mice

**Figure 5:**
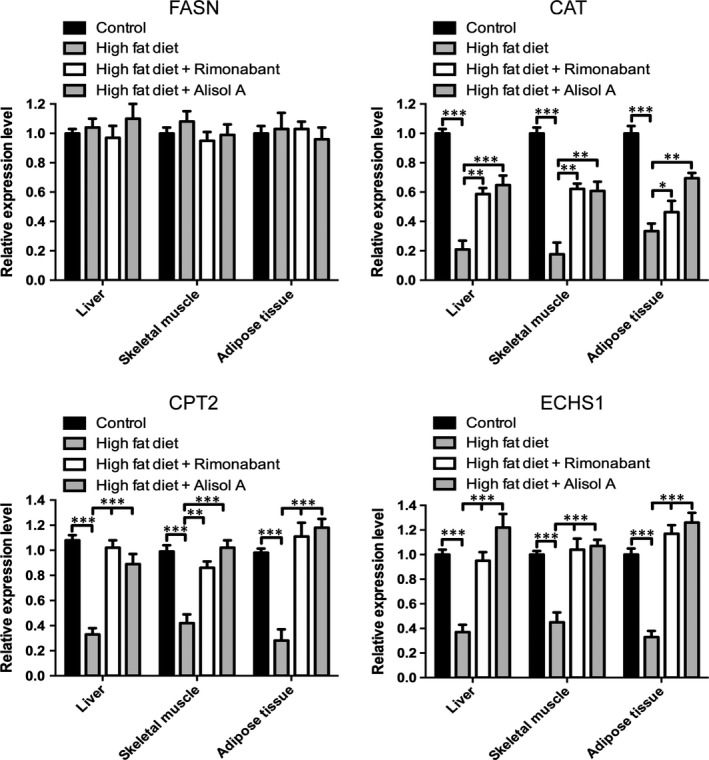
Alisol A improved damaged β‐oxidation in DIO mice. Relatively RNA expression levels of FASN(fatty acid synthase), CAT(carnitine acetyltransferase), CPT2(carnitine palmityltransferase II) and ECHS1(enoyl CoA hydratase) in extract of different group of mice adipose/liver/ skeletal muscle tissue. Data are the mean ± SD. NS = not significant; *P < 0.05, **P < 0.01, ***P < 0.001

Because alisol A did little influence on mice relative food intake, we thought the reduction in body weight induced by alisol A was accompanied by specific gene modulations. So we checked some important genes related to fatty acid synthesis and β‐oxidation using mice liver, skeletal muscle and adipose tissue (Figure 5). Results turned out that the mRNA level of FASN(fatty acid synthase) had no significant difference when comparing the HFA group to HF group. However, alisol A rescued the mRNA level of CAT(carnitine acetyltransferase), CPT2(carnitine palmityltransferase II) and ECHS1(enoyl CoA hydratase) which has decreased in DIO mice. These results suggested that alisol A may have no effect on fatty acid synthesis but it improved the damaged β‐oxidation process in DIO mice.

### 3.6 Alisol A activated the AMPK/ACC/SREBP‐1c signalling pathway in HDF‐induced obese mice

**Figure 6:**
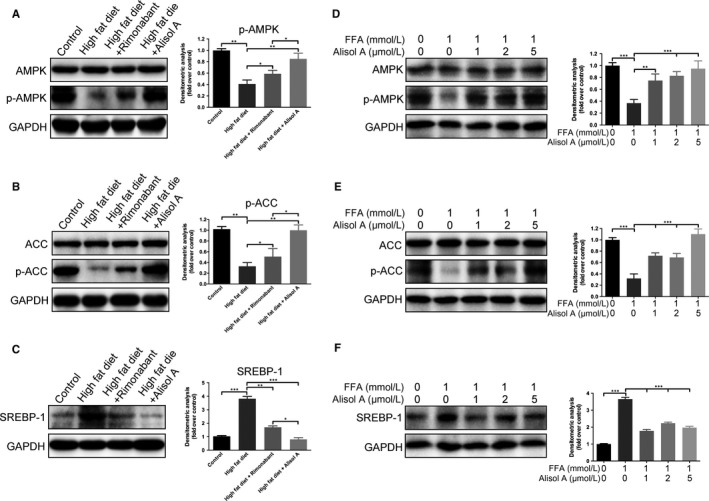
Alisol A attenuates HDF‐induced inhibition of AMPK/ACC pathway phosphorylation. (A, B) Protein levels of phosphorylated and total AMPK and ACC in liver tissues of the four groups of mice. C, Protein levels of SREBP‐1 in liver tissues of the 4 groups of mice. (D, E) Protein levels of phosphorylated and total AMPK and ACC in FAA untreated/treated HepG2 cells with addition of different concentration of alisol A. F, Protein levels of SREBP‐1 in FAA untreated/treated HepG2 cells with addition of different concentration of alisol A. Data are the mean ± SD. NS = not significant; *P < 0.05; **P < 0.01; ***P < 0.001

To further explore the underlying mechanism of how alisol A inhibited obesity and metabolic disorders, the signal transducer and the activator of transcription of the AMPK/ACC/SREBP‐1 signalling pathway were analysed in liver tissue. Western blot results indicated that p‐AMPK and p‐ACC were markedly down‐regulated in the HF group, while the total levels of AMPK and ACC remained unaffected (Figure 6A,B). Alisol A and rimonabant were able to restore the levels of p‐AMPK and p‐ACC, and this change was more pronounced in the HFA group compared with the HFR group (Figure 6A,B). By contrast, SREBP‐1c, an obesity‐related protein, was significantly up‐regulated in the HF group and significantly down‐regulated in the HFA group (Figure 6C). Not only in the liver, we also conducted the same Western blot using the skeletal muscle and adipose tissue, and we got almost the same results (Figure S2).

To make our results more convincing, we used HepG2 cell line to analyse the effect of alisol A in vitro. The results indicated that p‐AMPK and p‐ACC were markedly down‐regulated in HepG2 cells with free fatty acid (FFA) added group, while the total levels of AMPK and ACC remained unaffected, alisol A was able to restore the levels of p‐AMPK and p‐ACC in FFA added HepG2 cells, and this change was alisol A concentration dependent (Figure 6D,E). By contrast, SREBP‐1c was up‐regulated in the FFA added group and down‐regulated after alisol A addition(Figure 6F). All these data conducted with HepG2 cells suggested that AMPK/ACC/SREBP‐1c pathway participates in the regulation of obesity and metabolic disorders by alisol A.

### 3.7 Alisol A selectively bound to the catalytic region of AMPK

**Figure 7:**
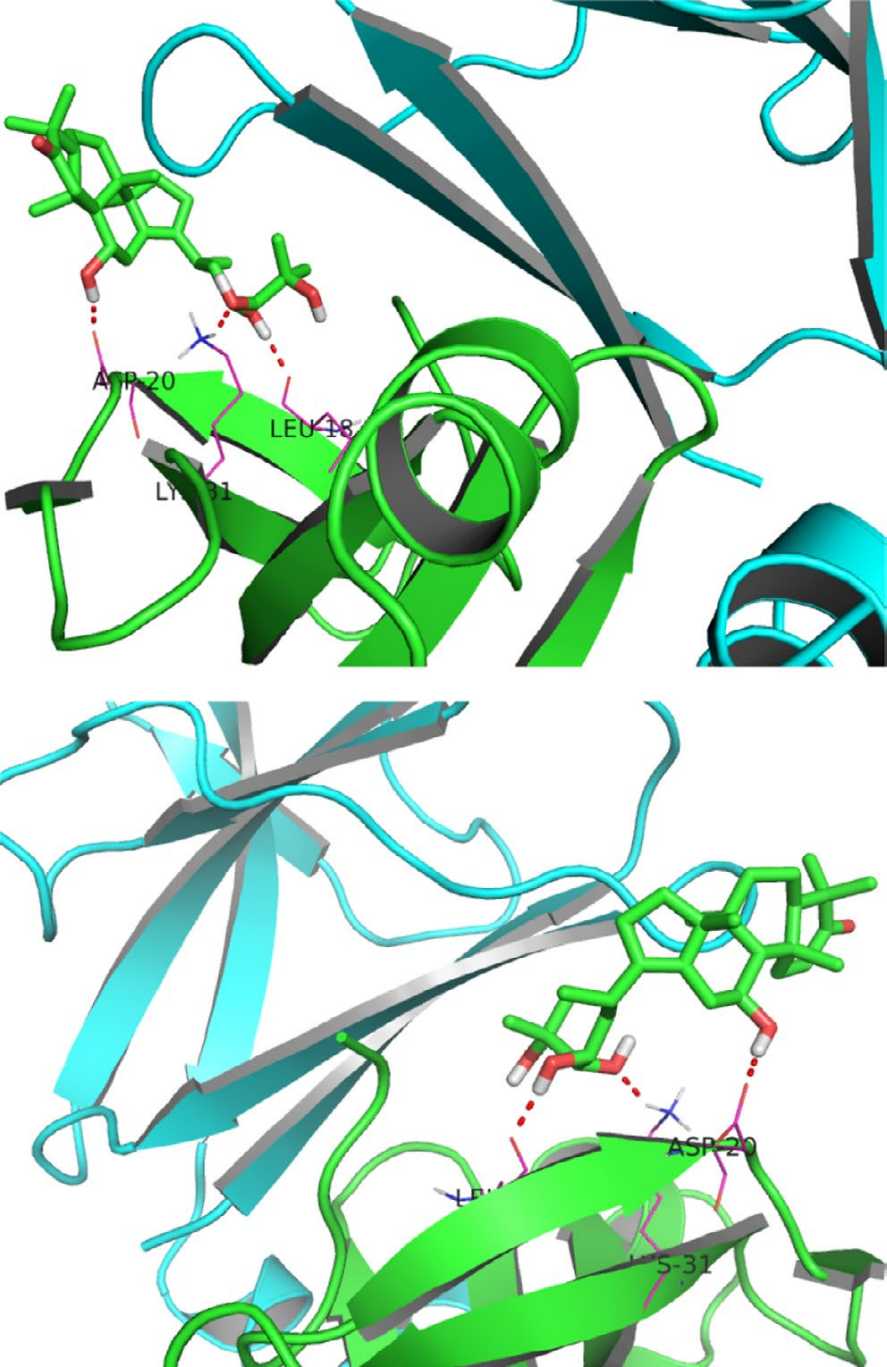
Alisol A binds to the catalytic region of AMPK. Computational docking simulation of the alisol A binding mode of the active conformation of AMPK. Carbon atoms are shown in blue and oxygen atoms in red. Hydrogen bonds are indicated by dashed lines

As we found that alisol A could activate the AMPK/ACC/SREBP‐1c pathway, we next aim to find out its specific target. Increasing evidence has shown that a variety of small molecule compounds may bind to AMPK and act as its activator.^27^ In light of this, we performed a computational protein‐molecule docking study in search of an interaction between alisol A and AMPK. The three‐dimensional interaction map showed that alisol A formed some favourable interactions with the active conformation of AMPK. As seen in Figure 7, alisol A can form three strong hydrogen bonds with the amino acid residues Leu18, Asp20 and Lys31 of AMPK. Due to these ligand‐protein interactions, alisol A could bind efficiently to the active conformation of AMPK, which suggested that alisol A may activate AMPK kinase activity.

## 4 DISCUSSION

Obesity is one of the most prevalent chronic metabolic disorders and one of the most important global public‐health challenges.^1^ This disease affects millions of people and has an incidence of approximately 13% of the adult population worldwide.^28^ Obesity is associated with a high risk of some chronic diseases, including type 2 diabetes,^29^ hyperlipemia and non‐alcoholic fatty liver disease (NAFLD),^30^, ^31^ posing a significant threat to human health. Despite the enormous efforts that have been made so far, current pharmacological approaches to morbid obesity are fairly limited.

In this study, we identified alisol A as a novel anti‐obesity treatment component that significantly decreased body weight and abdominal fat content in high‐fat‐diet‐induced obese mice. Abdominal fat accumulation is a particular characteristic of metabolic dysfunctions such as hyperlipemia and diabetes.^32^, ^33^ Consistent with the findings of previous studies using a high‐fat‐diet‐induced obesity model,^34^, ^35^ our data showed that excessive energy intake was highly associated with dyslipidemia and insulin resistance. Alisol A treatment was able to improve the parameters of hyperlipidemia, including plasma total cholesterol, triglycerides, LDL‐C, NEFA and FABP4. In line with our findings, Xu et al demonstrated that alisol‐based compounds could decrease triglyceride (TG) levels by increasing the activity of lipoprotein lipase (LPL), and the order of the intensity of the effect was alisol A>alisol B>alisol A 24‐acetate.^36^ In addition, we showed that alisol A improves glycometabolism and insulin sensitivity by reducing the increased insulin levels caused by a high‐fat diet. Obesity has been proven to be accompanied by inflammation in organs such as the liver and skeletal muscle, consequently promoting metabolic dysfunction and insulin resistance.^37^, ^38^ Considering that alisol A has been shown to have anti‐inflammatory^20^ properties, our results also suggested that it could alleviated inflammation in adipose tissue of DIO mice, we supposed that the metabolic improvement effects of alisol A may be partially due to it's suppression of inflammatory damage.

One of the most deteriorative effects of obesity is ectopic lipid deposition, which leads to pathological changes in internal organs.^39^ In obesity, a greater amount of fatty acids flow into hepatocytes and lipid oxidation is reduced.^40^ Moreover, hepatocytes exhibit increased lipid synthesis and glycolysis, resulting in the typical accumulation of fat in the liver.^41^ In this study, we showed that with the increased levels of hyperlipidemia in high‐fat‐diet‐fed mice, excess lipids were stored in liver cells and formed fat vacuoles (liposomes). However, these pathological alterations could be attenuated by treatment with alisol A. In hepatic steatosis, excessive lipid deposits lead to hepatocellular injury^42^; moreover, a state of inflammation and accumulation of reactive oxygen species (ROS) could impair liver function.^43^ We found that alisol A markedly decreased serum levels of ALT and AST, suggesting that alisol A treatment had no hepatotoxic effects and was able to reverse obesity‐associated hepatocellular injury.

We found that alisol A attenuates HFD‐induced obesity and metabolic disorders and alisol A could partialy rescue the damaged β‐oxidation in DIO mice, which is an important part in treating obesity.^44^ Thus, we wanted to investigate the underlying mechanism. The AMPK/ACC pathway plays an important role in the regulation of fatty acid metabolism.^45^, ^46^ AMPK is a sensor of cellular energy charge and a ‘metabolic master switch’.^47^ When activated by ATP depletion, it switches off ATP‐consuming processes, while switching on catabolic pathways that generate ATP.^48^ AMPK mediates the suppression of lipogenic gene expression involved in triglyceride synthesis and accumulation via the phosphorylation of ACC.^49^ In this study, we found that the levels of AMPK and ACC were significantly diminished in the liver tissues of high‐fat‐diet‐induced obese mice, suggesting an increase in AMPK/ACC activity and liver lipogenesis. Interestingly, liver p‐AMPK and p‐ACC of obese mice were markedly upregulated by alisol A treatment. These findings are consistent with those of a previous study in which the triterpenoid isolated from Rhizoma Alismatis ameliorated non‐alcoholic steatohepatitis through the AMPK pathway.^25^ Genes involved in liver glycolysis and lipogenesis are regulated at the transcriptional level through the transcription factor SREBP‐1c.^50^ Moreover, the phosphorylation of AMPK suppressed the cleavage and nuclear translocation of SREBP‐1c and led to a reduction in lipogenesis and lipid accumulation.^51^ Our results showed that the upregulation of SREBP1c expression triggered by a high‐fat diet was diminished in the presence of alisol A both in vivo and in vitro. These data were also parallel to the degree of decreased hyperlipidemia and hepatic steatosis and improved liver function indices ALT and AST, suggesting that the AMPK/ACC/SREBP‐1c pathway likely participates in mediating the anti‐obesity and metabolic disorder improvement properties of alisol A.

In summary, our study demonstrated that alisol A effectively attenuated HFD‐induced obesity, suppressed hepatic steatosis and improved lipid and glucose metabolism, and improved damaged β‐oxidation in DIO mice. In addition, it alleviated inflammation state in DIO mice adipose tissue. Our in vivo and in vitro data also revealed that the pharmacologic action of alisol A may be mediated through AMPK/ACC/SREBP‐1c pathway activation. From these studies, we considered that alisol A is a highly promising component that could serve as a treatment for obesity and obesity‐related metabolic disorders.

## CONFLICT OF INTEREST

The authors declare that they have no conflict of interest.

## Data Availability

The data that support the findings of this study are available from the corresponding author upon reasonable request.
