# Supplementary material for: Alisol A attenuates high‐fat‐diet‐induced obesity and metabolic disorders via the AMPK/ACC/SREBP‐1c pathway
Source: J Cell Mol Med. 2019 May 29;23(8):5108–18. doi: 10.1111/jcmm.14380 (PMC6653754; doi:10.1111/jcmm.14380)
Supplement: Supplementary file 1 [file JCMM-23-5108-s001.docx]

Supplementary Figure S1. The chemical structure of alisol A.

Supplementary Figure S2. Alisol A attenuates HDF-induced inhibition of AMPK/ACC pathway phosphorylation in white adipose tissue and skeletal muscle. (A, B, C) Protein levels of phosphorylated and total AMPK, ACC and Protein levels of SREBP-1 in white adipose tissue of the 4 groups of mice. (D, E, F) Protein levels of phosphorylated and total AMPK, ACC and Protein levels of SREBP-1 in skeletal muscle of the 4 groups of mice. Data are the mean ± SD. NS = not significant; **p* < 0.05; ***p* < 0.01; ****p* < 0.001.
